# Effects of Nursing Education Using Films on Perception of Nursing, Satisfaction With Major, and Professional Nursing Values

**DOI:** 10.1097/JNR.0000000000000433

**Published:** 2021-04-20

**Authors:** Hyangjin PARK, Haeryun CHO

**Affiliations:** 1 PhD, RN, Associate Manager, Department of Prevention and Public Relations & Research and Development Team, Korea Center on Gambling Problems, Seoul, Republic of Korea; 2 PhD, RN, Associate Professor, Department of Nursing, Wonkwang University, Iksan, Republic of Korea.

**Keywords:** films, nursing education, nursing students, satisfaction

## Abstract

**Background:**

Cinenurducation, a film-based approach to nursing education that incorporates student-centered, problem-solving, experiential, and reflective learning strategies, allows students to experience a variety of indirect experiences and improves critical thinking and self-reflection through discussion.

**Purpose:**

The aims of this study were, first, to employ a cinenurducation approach to help instill a proper professional nursing identity in second-year nursing students and, second, to examine the effects of this approach on the perception of nursing, satisfaction with major, and professional nursing values of the participants.

**Methods:**

An experimental, pretest-and-posttest design was used to test the primary variables, including perception of nursing, satisfaction with major, and professional nursing values. The nursing educational program was developed based on the learning concepts of cinenurducation and the core concepts of nursing. The program, which included six films, addressed the following concepts: *Me Before You* (problem solving and professionalism), *Testament of Youth* (nursing management and professionalism), *Girl, Interrupted* (interpersonal skills and nursing knowledge), *Hungry Heart* (interpersonal skills and problem solving), *Iris* (nursing knowledge and problem solving), and *Chronic* (nursing knowledge and cooperation). The experimental group (*n* = 14) participated in the 8-week educational program, and the control group (*n* = 15) did not.

**Results:**

Perception of nursing, satisfaction with major, and professional nursing values all improved significantly more in the experimental group than in the control group, with large effects observed.

**Conclusions:**

Cinenurducation is an effective approach to promoting professional nursing identity in nursing students. Educators should incorporate films into nursing education. In addition, nursing education should incorporate a variety of educational materials to provide students with opportunities for reflective learning.

## Introduction

Professional nursing identity refers to the awareness of the functions and roles that professional nurses are expected to perform in clinical contexts (Dadich & Doloswala, 2018). Developing a positive professional nursing identity is critical for nursing undergraduate students (Browne et al., 2018). Nursing students who are not satisfied with their major may neglect their studies, hold a negative perception of nursing (Admi et al., 2018; Feng et al., 2016), and fail to establish a proper professional nursing identity, which may negatively affect their development of professional competencies (Mazhindu et al., 2016). Having a proper professional nursing identity enables nurses to examine and judge clinical situations comprehensively (Heldal et al., 2019). Thus, nursing students must be provided with a nursing education that promotes the development of a proper professional nursing identity. In other words, professional nursing identity may be connected to perception of nursing, satisfaction with major, and professional nursing values (Woo & Park, 2017).

Perception of nursing refers to beliefs regarding the traditional, social, and professional impressions of nurses and nursing prospects (de Braganca & Nirmala, 2018; Emeghebo, 2012). Nursing students who hold positive perceptions of nursing display high levels of self-esteem, professional intuition, and professional identity. Furthermore, these students are highly likely to become good nurses in the clinical field (de Braganca & Nirmala, 2018; Woo & Park, 2017). Therefore, developing a positive perception of nursing is essential to improving nursing identity.

Satisfaction with major refers to students' satisfaction with meeting their expectations related to their academic major. A good understanding of the career associated with one's major increases this satisfaction (Feng et al., 2016). In particular, nursing students' satisfaction with their major promotes dedication to studies, helps them develop positive values about their major, and affects their nursing-related knowledge and attitudes (Feng et al., 2016). Thus, nursing students with high levels of satisfaction with their major are more likely to become good nurses with a clear nursing identity (Admi et al., 2018).

Professional nursing values are the overall beliefs that encompass views on nurses' activities and roles as well as opinions on nurses as professionals (Ayla et al., 2018; Bijani et al., 2019). As the starting point of nursing students' development into professional nurses, professional nursing values relate directly to excellent nursing (Bijani et al., 2019; Pickles et al., 2019). These values are important because they improve the quality of nursing, understanding of patients, and job satisfaction (Bijani et al., 2019; Parandeh et al., 2014).

Positive professional values are developed continuously in nursing students through their education and learning experience (Kantek et al., 2017; Kaya et al., 2017; Parandeh et al., 2014). These values affect their satisfaction with major and their perception of nursing (Ahn & Song, 2015; Cho & Kim, 2016; Lim & Jo, 2016). Having well-established professional nursing values helps nurses make better decisions and develop a positive professional nursing identity, which can help them avoid moral pain and provide quality care (Parandeh et al., 2014; Posluszny & Hawley, 2017; Woo & Park, 2017).

Films have been reported to be effective in helping students experience a variety of learning including emotions, feelings, knowledge, actions, skills, and attitudes (J. Oh, Kang, et al., 2012; J. Oh & Steefel, 2016; Zeppegno et al., 2015). When selecting a film, the film content must match the learning objectives and, if students are able to understand and discuss the film, then any kind and characteristic of the film can be used (J. Oh, Kang, et al., 2012; J. Oh & Steefel, 2016). Therefore, using films as an educational tool helps students understand nursing situations that they cannot otherwise fully experience (J. Oh, Shin, et al., 2012; J. Oh & Steefel, 2016).

Cinenurducation is an approach to nursing education that has recently attracted significant attention (Klemenc-Ketis & Kersnik, 2011; J. Oh, Kang, et al., 2012; Zeppegno et al., 2015). Cinenurducation, based on the experiential learning theory of Kolb (1984), involves the student-centered, problem-solving, experiential, and reflective types of learning (J. Oh, Kang, et al., 2012; J. Oh & Steefel, 2016). One advantage of this educational method is that it provides opportunities to discuss and debate issues raised through films. The film format allows learners to discuss issues concisely and calmly, which promotes students' problem-solving and creative thinking capabilities (Kim, 2014; J. Oh, Kang, et al., 2012). In addition, studies have shown that students clarify their knowledge and attitudes by watching films, discussing films in group settings, and talking about one another's reactions, thereby developing critical thinking and self-reflection (J. Oh, Shin, et al., 2012; J. Oh & Steefel, 2016). In other words, cinenurducation stimulates the visual areas of the learner's brain, allowing learners to experience a situation, emotionally empathize, and formulate solutions while actively participating in the class and discussing solutions (Edmonds, 2011; Kolb, 1984; J. Oh, Kang, et al., 2012).

Research related to cinenurducation has focused on understanding patients and health promotion in nursing (Briggs, 2011), multicultural nursing and multicultural competence enhancement (Edmonds, 2011), and shedding light on mental illness and mental health nursing (Zauderer & Ganzer, 2011). However, in these studies, students were not able to gain a comprehensive understanding of nursing because of the reliance on using film clips and excerpted scenes rather than complete film content. Films may need to be viewed in full to maximize learning efficacy. J.-A. Oh (2010) suggested that learners should watch films in their entirety to gain a comprehensive understanding of the situation and content of the main character, which will maximize opportunities for discussion and creative learning. Therefore, the educational effects of cinenurducation in which full film content is used remain to be verified.

Some studies have indicated that third-year nursing students with clinical practice experience tend to have a negative perception of nursing, low satisfaction with their major, and inadequate professional nursing values (Emeghebo, 2012; Lee, 2004; Woo & Park, 2017). These tendencies have been attributed to their experiencing clinical practicum before their professional nursing identity had fully formed. Therefore, this study was designed to use films in nursing education to establish a proper professional nursing identity in second-year nursing students and to examine the effects of this education on perception of nursing, satisfaction with major, and professional nursing values. The research questions addressed in this study were as follows: (a) “Did the intervention improve perception of nursing?”, (b) “Did the intervention improve nursing major satisfaction?”, and (c) “Did the intervention improve nursing professional values?”

### Conceptual Framework

**Figure 1.**
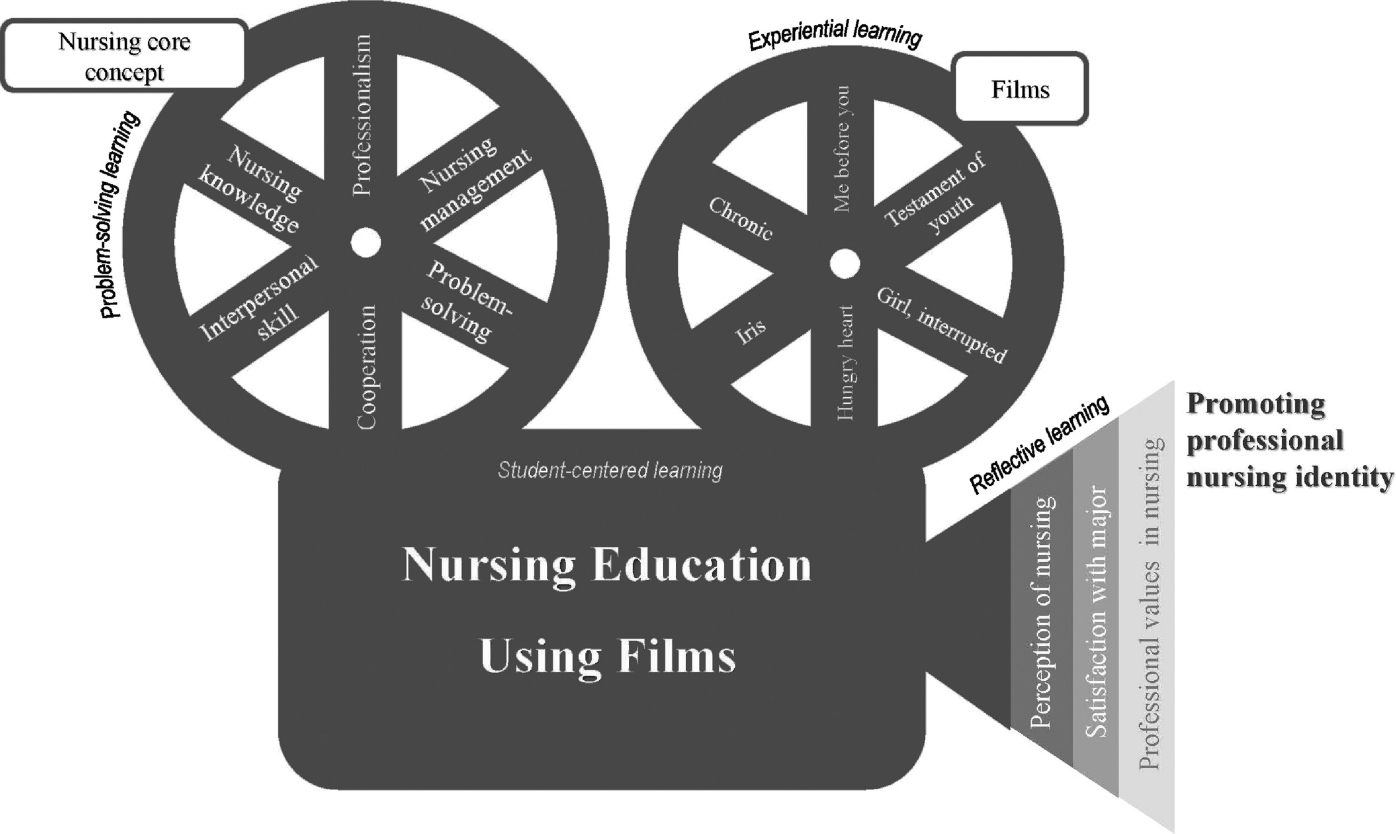
Conceptual Framework of this Study Based on Conceptual Metaphor of Cinenurducation by J. Oh, Kang, et al. (2012)

The conceptual framework in this study is shown in Figure 1. The intervention method in this study was developed using the learning concepts of cinenurducation (J. Oh, Kang, et al., 2012), which include student-centered, problem-solving, experiential, and reflective learning. In terms of student-centered learning, the learning objectives were introduced briefly before film viewing, and the wide degree of freedom allowed to the participants to analyze and reflect was guaranteed. In terms of problem-solving learning, students were introduced to discussion topics related to the core concepts of nursing, which were communicated through various situations in the films, before watching the film (Park et al., 2013). Furthermore, six films related to nursing core concepts were used to provide experiential learning, which induced indirect experiences of various situations. Finally, in terms of reflective learning, students shared their feelings and engaged in a discussion on the topics raised after each film was screened.

To promote professional nursing identity by improving perception of nursing, satisfaction with major, and professional nursing values, the contents of nursing education were organized using the nursing core concepts proposed by Park et al. (2013), which represent the most common and basic nursing tasks in clinical settings.

## Methods

### Study Design

**Figure 2.**
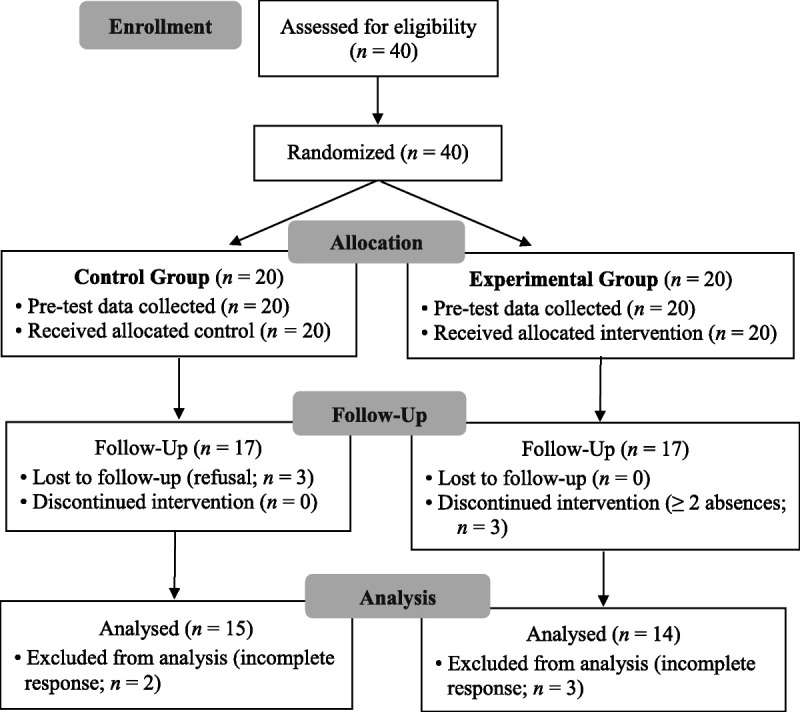
Participant Recruitment Process

An equivalent control group was used in this study. A pretest/posttest, nonsynchronized design was used to examine the effect of the intervention on participants' perception of nursing, satisfaction with major, and professional nursing values (Figure 2).

### Participants and Setting

The participants in this study were nursing students in their second year of a 4-year undergraduate program of a university located in Iksan City. In undergraduate nursing programs in South Korea, first-year students take an introductory course on nursing, second-year students take a basic major course and laboratory practicum, and third- and fourth-year students take a major course and clinical practicum. Nursing students are expected to establish their professional nursing identity before completing their nursing major course (Ayla et al., 2018). Therefore, the inclusion criteria for participants were (a) second-year nursing student and (b) voluntarily agreeing to participate, whereas the exclusion criteria were (a) having previously received nursing education via artwork such as films, paintings, or literature or (b) being absent from the intervention more than twice.

The educational intervention in this study was formatted as an extracurricular class that was not compulsory and was not graded. The researchers publicly posted the syllabus, overall purpose of the research, and participant criteria, and 40 students applied as volunteers to participate in the class and research.

G*Power 3.1.3 (Faul et al., 2007) was used to calculate the sample size required (significance level = .05, effect size = 1.0, power = .80). The effect size reported in previous studies (Kim, 2014; Zeppegno et al., 2015) was 1.0. As the required sample size estimated for this study was 14 nursing students per group, the total number of participants required was 28. After estimating the potential dropout rate, 20 students were recruited per group. In the control group, five participants withdrew, including three who did not attend the posttest and two who provided invalid answers on the questionnaire. In the experimental group, six participants withdrew, including three who were absent from two or more sessions and three who provided invalid answers on the questionnaire (Figure 2). Thus, the data of 29 participants (experimental group = 14, control group = 15) were available for analysis, which met the required number of subjects for this study.

### Nursing Education Using Films

Films were selected for the intervention that were appropriate to the purpose of this study and in line with the criteria described by J. Oh, Shin, et al. (2012). These criteria are as follows: (a) absent of controversial issues related to sexuality and addressing only generally accepted concepts related to culture, emotion, ideology, or philosophy; (b) a popular genre (not an educational material or a documentary) that may be watched in a relaxed manner; and (c) having the strong potential to generate sufficient discussion questions on the nursing core concepts proposed by Park et al. (2013). Eleven films were initially selected based on literature reviews and data from previous research and on a list of films screened at a nursing film festival held by the Korean Nurses Association (2015). Of these 11 films, three were not available on digital video disk and were excluded. After watching the remaining eight films, the researchers excluded two films with content deemed as difficult for students to understand. The final six films were used in this study. The researchers arranged the film screenings based on the nursing core concepts, from general to specific contents, and then developed discussion topics. The six films and the concepts addressed were as follows: *Me Before You* (problem solving and professionalism), *Testament of Youth* (nursing management and professionalism), *Girl, Interrupted* (interpersonal skills and nursing knowledge), *Hungry Heart* (interpersonal skills and problem solving), *Iris* (nursing knowledge and problem solving), and *Chronic* (nursing knowledge and cooperation; Table 1).

**Table 1.** Contents of Nursing Education Using Films

Session	Nursing Core Concept	Content	Film	Running Time (Min)	Country (Years)	Discussion Topics	Method (Time)
1	–	Orientation	–	–	–	• Information about this course and schedule	• Orientation (5 min) -Introduce movie -Discussion topics • Movies (90–120 min) • Break (10 min) • Discussion (30–40 min) -Sharing thoughts on topics • Wrap-up (10 min) - Feedback
2	• Problem solving	Critical thinking	*Me Before You*	110	United States (2016)	• Discussion about bioethics and ethical concepts (principles of autonomy) • What is well-dying? • Pros and cons of death with dignity as a health professional	
	• Professionalism	Ethical issue					
3	• Nursing management	Leadership	*Testament of Youth*	129	England (2014)	• The role of nurses in contributing to national crises • Discussion about ethical concepts (principles of precedence, justice, and sincerity) • Ethical dilemma: national profit versus maintenance of individual life	
	• Professionalism	Ethical value oriented					
4	• Interpersonal skill	Communication	*Girl, interrupted*	127	United States (1999)	• Understanding the patients with mental health problems • Critique of therapeutic communication in film • Cases related to mental health such as depression, anxiety, and social adaptation in film	
	• Nursing knowledge	Mental health					
5	• Interpersonal skill	Patient understanding	*Hungry Heart*	112	United States (2014)	• Understanding the various health beliefs of family members • The influence of family belief and value on health • Applying the nursing process for family health	
	• Problem solving	Nursing process					
6	• Nursing knowledge	Dementia	*Iris*	90	United States (2001)	• What is dementia? • The influence of dementia on the family • Applying the nursing process for dementia patients and their family	
	• Problem solving	Nursing process					
7	• Nursing knowledge	Chronic disease	*Chronic*	94	Mexico (2015)	• Understanding chronic diseases and hospice care • What is patient-centered care? • Establishing the therapeutic relationship between nurses and patients as professionals	
	• Cooperation	Patient-centered care					
8		Wrap up	–	–	–	• Sharing feelings about this course	

***Note.*** Min/min = minutes.

J. Oh and Steefel (2016) suggested that cinenurducation allows nursing students to approach discussion topics from various angles, helping them better understand the characters and background of the film. Thus, in this study, the films were screened in full. The researchers identified topics and set up discussions to enable the students to deduce the core concepts of nursing after viewing the films. For example, the main character of the film *Chronic*, a hospice nurse, shared a relationship with his patients similar to that of a wife or siblings. Moreover, a patient with cancer demanded that he kill her. The discussion topics for this film, which were selected to help students derive nursing core concepts, included “What are chronic diseases and what is your understanding of hospice care?” and “What is patient-centered care, and what is an appropriate therapeutic relationship between nurses and patients?”

The intervention was conducted once per week for 8 weeks. Each session took approximately 3 hours and was organized as follows: 5 minutes of orientation, 90–120 minutes of film viewing, 10 minutes of break time, 30–40 minutes of discussion to share feelings and thoughts on the discussion topics, and 10 minutes of feedback and summary. The content validity of this program with regard to the core concepts of nursing and the discussion topics were verified twice by three professors of nursing after watching the six films together.

### Measurements

#### Demographic variables

To test homogeneity, information on gender, age, satisfaction with major, perceived difficulty of major, and motivation for selecting major was collected from the participants. A 5-point Likert scale was used to score satisfaction with major, with higher scores indicating greater satisfaction. Perceived difficulty of major included lecture class, practice class, others, or none. Motivation for selecting major included suggestion of acquaintance, fitting aptitude, or high employment rate.

#### Perception of nursing

The 20-item checklist of Kang et al. (2003) was used to measure perception of nursing. Of these 20 items, six were related to professional perception, six were related to traditional perception, three were related to forecast about nursing, and five were related to social perception. All items were scored using a 5-point Likert scale, with higher scores indicating a more positive perception of nursing. The measurement reliability, as measured using Cronbach's alpha, was .94 in Kang et al. and .78 in this study.

#### Satisfaction with major

Satisfaction with major was measured using an 18-item scale that was revised by Lee (2004) from a 34-item instrument by Ha (1999). Items were measured using a 5-point Likert scale, with higher total scores indicating greater satisfaction. Satisfaction with major included general satisfaction about nursing, satisfaction with social awareness related to the nursing major, content satisfaction with the classes of the nursing major, and satisfaction with the relationship between professors and students (Lee, 2004). The measurement reliability, as measured using Cronbach's alpha, was .94 in Lee and .86 in this study.

#### Professional nursing values

To measure professional nursing values, the 29-item scale developed by Yeun et al. (2005) was used. This scale covers the five dimensions of professional self-concept (nine items), social recognition (eight items), nurse professionalism (five items), practical nursing role (four items), and nursing identity (three items), and items are scored using a 5-point Likert scale, with higher scores indicating stronger nursing professional values. The measurement reliability, as measured by Cronbach's alpha, was .92 in Yeun et al. and .85 in this study.

### Data Collection and Ethical Considerations

This study was conducted after approval for the ethical consideration of participants was obtained from the institutional review board of the researchers' institution (IRB No. WKIRB-201703-SB-015). For blinding purposes, the first author and second author were separately involved, respectively, in collecting the study data and conducting the intervention. Using SPSS, the participants were randomly assigned to the experimental and control groups in two classes of 20 students. Only the data collector knew the designation of each group to ensure that the instructor and the participants remained blinded.

Data were collected from August 28 to December 13, 2017. Pretest and posttest data were collected first from the control group and then from the experimental group. The control group took the pretest between August 28 and 30, 2017, with the posttest conducted between October 18 and 20, 2017, after conclusion of the intervention. The experimental group took the pretest on October 25, 2017, with the posttest conducted on December 13, 2017, after conclusion of the intervention.

Informed consent forms, general information on the study, study research aims, and information on voluntary consent/withdrawal were provided to potential participants. After providing informed consent, the participants were enrolled and completed the questionnaires, which took about 5 minutes. A small gift was presented to each participant upon questionnaire completion.

The control group had received no film teaching or other supplemental teaching interventions. For ethical considerations, the intervention was given to the control group after all study data had been collected.

### Data Analysis

The collected data were analyzed using IBM SPSS Statistics 24.0 (IBM, Inc., Armonk, NY, USA). The general characteristics, perception of nursing, satisfaction with major, and professional values were estimated by number, percentage, mean, and standard deviation. The skewness of these variables was between −0.73 and 0.52, and kurtosis was between −0.57 and 0.33. As the skewness and kurtosis were both < ±1.965, the collected data were interpreted as normally distributed. Homogeneity between the experimental and control groups was verified using a chi-square test and an independent *t* test. To identify the effects of nursing education using films, an independent *t* test was used, and the effect size was calculated using Cohen's *d* formula. Dependent variables that were not homogeneous in the pretest were controlled by covariance, analyzed using analysis of covariance, and were assessed for effect size using the eta-squared formula.

## Results

### Homogeneity Test

No significant difference was observed between the two groups in terms of gender, age, general satisfaction with major, perceived difficulty of their major, motivation for selecting major, satisfaction with major, or professional nursing values (Table 2). However, a significant intergroup difference in the pretest score of perception of nursing was found and subsequently analyzed by controlling covariance to verify the effect.

**Table 2.** Intergroup Test for Homogeneity (*N* = 29)

Characteristic	Experimental Group (*n* = 14)		Control Group (*n* = 15)		*t*/χ^2^	*p*
	*n*	%	*n*	%		
Gender					3.12	.077
Male	0	0.0	3	20.0		
Female	14	100.0	12	80.0		
Age (years; *M* and *SD*)	19.79	0.80	19.60	0.83	0.61	.545
General satisfaction with major (*M* and *SD*)	3.64	0.63	3.87	0.92	0.76	.454
Perceived difficulty of major					3.42	.331
Lecture class	7	50.0	12	80.0		
Practice class	2	14.3	1	6.7		
Others	1	7.1	1	6.7		
None	4	28.6	1	6.7		
Motivation for selecting major					4.96	.084
Suggestion of acquaintance	4	28.6	6	40.0		
Fitting aptitude	1	7.1	5	33.3		
High employment rate	9	64.3	4	26.7		
Prescore (*M* and *SD*)						
Perception of nursing	3.59	0.27	3.89	0.27	2.91	.007
Satisfaction with major	3.91	0.33	3.97	0.40	0.43	.673
Professional values in nursing	3.49	0.34	3.65	0.32	1.33	.195

### Effects of Nursing Education Using Films

The effects of nursing education using films are shown in Table 3. The independent *t* test of the mean difference between posttest and pretest scores showed that the intervention had a significant effect on satisfaction with major (*t* = 2.59, *d* = 0.97, *p* = .018) and professional nursing values (*t* = 2.92, *d* = 0.93, *p* = .007) in the experimental group. Analysis of covariance, which controlled for the perception of nursing pretest scores by covariance, showed that the intervention had a significant effect on perception of nursing (*F* = 6.88, η^2^*=* 0.55, *p* = .014), with large effects observed (Cohen, 1988).

**Table 3.** Effects of Nursing Education Using Films (*N* = 29)

Variable	Experimental Group (*n* = 14), *M* ± *SD*		Control Group (*n* = 15), *M* ± *SD*		*t*	*p*	Cohen's *d*
	Pretest	Posttest	Pretest	Posttest			
	Difference Between Posttest and Pretest		Difference Between Posttest and Pretest				
Perception of nursing	3.59 ± 0.27	4.06 ± 0.57	3.89 ± 0.27	3.78 ± 0.27			
	0.48 ± 0.56		−0.10 ± 0.21		6.88 ^a^	.014	0.55 ^b^
Satisfaction with major	3.91 ± 0.33	4.27 ± 0.35	3.97 ± 0.40	3.95 ± 0.39			
	0.37 ± 0.50		−0.02 ± 0.23		2.59	.018	0.97
Professional nursing values	3.49 ± 0.34	3.88 ± 0.46	3.65 ± 0.32	3.60 ± 0.38			
	0.40 ± 0.48		−0.05 ± 0.34		2.92	.007	0.93

^a^ Analysis of covariance (pretest score). ^b^ Eta squared (η^2^).

## Discussion

This study was conducted to verify the effect in nursing students of a film-based nursing educational intervention on perception of nursing, satisfaction with major, and professional nursing values. To evaluate the effects of the program, the changes in pretest and posttest results were examined between the experimental and control groups.

After the completion of the intervention, a larger increase in the mean score for perception of nursing was observed in the experimental group than in the control group, with a large effect size of 0.55 (Cohen, 1988). This result supports that nursing students' perception of nursing may be improved by educating them about nursing knowledge, skills, attitudes, beliefs, values, and ethical standards (Ayla et al., 2018; de Braganca & Nirmala, 2018). In particular, nursing students who meet professional nurses during hospital visits or clinical practice may be highly affected by the experience (de Braganca & Nirmala, 2018). In this study, participants in the experimental group engaged in 30–40 minutes of group discussion on each film with an instructor who was an expert in nursing. This interactivity provided opportunities for participants to share opinions with a nursing expert on related knowledge, ethical dilemmas, nursing roles, and beliefs and values related to patients and their families. Regardless of teaching style, an instructor's guidance and questions during discussion or reinforcement of students' opinions may help students think deeper about the core concepts that underlie nursing. The discussion, participated in jointly by the instructor and students, contributed to enhancing the participants' perception of nursing. The learning experience of watching and discussing films in groups has been reported to improve the effectiveness of reflective learning through the sharing of reactions and opinions with others (J. Oh, Shin, et al., 2012; J. Oh & Steefel, 2016). In this study, the film-based nursing education intervention effectively improved the participants' perception of nursing because the group and discussion activities with the instructor were excellent venues for promoting participant reflection. Therefore, nursing education using a variety of teaching methods is necessary to allow students to reflect fully on their learning.

Furthermore, satisfaction with major was found to have improved significantly in the experimental group, with a large effect size of 0.97 (Cohen, 1988). As few studies have focused on whether films for nursing students affect satisfaction with major, it is difficult to conduct a comparison of other study results with this study. The core concepts of nursing, including professionalism, nursing management, problem solving, cooperation, interpersonal skills, and nursing knowledge, were dealt with in this intervention, with nursing students having the opportunity to think deeply about their perceptions of nursing science. In previous studies, clear awareness of nursing science has been found to relate to satisfaction with the nursing major (Ahn & Song, 2015; Cho & Kim, 2016; Kaya et al., 2017). In this study, the intervention helped the participants establish the value of nursing science, which may promote nursing major satisfaction. In addition, this study, which applied cinenurducation to promote student-centered, experiential, and reflective learning, empowered the participants to discuss freely on nursing topics with instructors rather than limiting them to listening to traditional lectures (J. Oh, Kang, et al., 2012; Zeppegno et al., 2015). This aspect may have had a positive effect on nursing major satisfaction by building closer relationships between instructors and students. Many studies suggest that nursing students should be provided with various experiences that promote increased satisfaction with their major, because students with higher levels of satisfaction showed higher academic achievement and nursing professionalism (Bijani et al., 2019; Pickles et al., 2019; Woo & Park, 2017). Therefore, the intervention in this study may be used as an effective educational strategy to increase nursing students' satisfaction with their major, which may ultimately improve their professional nursing identity.

In addition, nursing education using films was found to be effective in improving professional nursing values, with a large effect size of 0.93 (Cohen, 1988). According to Zeppegno et al. (2015), film-based education for medical students positively affects attitudes toward psychiatry including social distance from a patient with a mental disorder and interpersonal reactivity, such as dispositional empathy. Klemenc-Ketis and Kersnik (2011) reported that education using films positively influences communication, empathy, and attitude in medical students. The results of this study are similar. The viewing of films in their entirety helped the participants better understand nursing situations (J.-A. Oh, 2010; Zeppegno et al., 2015). In-depth discussions after watching the films helped the participants clarify their attitudes and knowledge and reinforced their professional nursing values while accepting others' opinions (J. Oh & Steefel, 2016). In this study, the indirect experience of nursing scenarios through the films, including situations that promoted problem-solving and reflective learning, gave the participants a deeper understanding of various situations. Moreover, the participants discussed topics related to nursing core concepts and shared opinions, which helped enhance their professional nursing values. Therefore, the intervention in this study may be an effective strategy for developing positive professional nursing values and, subsequently, improving professional nursing identity in nursing students.

Nursing education using films was developed based on the learning concepts of cinenurducation suggested by J. Oh, Kang, et al. (2012) and the core concepts of nursing (Park et al., 2013). The films screened in this study were selected to introduce discussion topics related to the core concepts of nursing. Because the selected film reflects the core concept of nursing, nursing education using films can be regarded as effective in promoting professional nursing identity. The purpose of watching films was to engage in indirect experiential learning on the core concepts of nursing. Participants watched films in their entirety, which made it easier to understand the situations, characters, and contexts in their entirety. To facilitate understanding of the nursing core concepts in nursing students, discussions with an instructor and peers were conducted after watching the films. An educator who understands the nursing core concepts participated in the discussion, providing a role model for the nursing students. Furthermore, even films, which could be difficult at the level of second-year students, were able to reflect through discussions in which all peer students participated.

Although this program helped nursing students understand the content of the films and organize their opinions, one limitation was the length of time required for each session. Therefore, a teaching method that requires less time per session is needed. Educators may provide orientation and discussion topics on the films via online sources, and then students could watch the film in advance and discuss the topics offline. Meanwhile, the films in this study were selected from Western countries only. Thus, the selection lacked consideration of possible differences/discrepancies between Eastern and Western cultural settings. Therefore, further research is needed to account for cultural contexts in films. In addition, as this study was conducted on second-year nursing students, the nursing professional identity formed before the clinical practicum may conflict with the experience in the clinical setting. Therefore, longitudinal research should be conducted to confirm the persistence of intervention effects through graduation.

### Conclusions

This study was conducted to verify whether nursing education using films affected the perception of nursing, satisfaction with major, and professional nursing values in nursing students. The results showed that the intervention was effective in these three aspects. Therefore, it is suggested that the intervention developed in this study be used as a strategy to improve the professional nursing identity of nursing students.

On the basis of this study, the implications of nursing education in undergraduate programs are as follows. First, nursing students' indirect experience through using films may promote learning of the core concepts of nursing. Therefore, educators should actively attempt nursing education using films. Second, this intervention was meaningful in terms both of using films as an educational resource and of promoting reflective learning through in-depth discussions. In the future, nursing education should be designed to provide students with opportunities for reflective learning using various educational materials. Finally, to help better establish the professional nursing identity of undergraduate students, educators should offer various supplementary courses such as the intervention attempted in this study in addition to the standard curriculum.

## References

[bib1] Admi H. Moshe-Eilon Y. Sharon D., & Mann M. (2018). Nursing students' stress and satisfaction in clinical practice along different stages: A cross-sectional study. *Nurse Education Today*, 68, 86–92. 10.1016/j.nedt.2018.05.027 29894915

[bib2] Ahn T., & Song Y. A. (2015). Affecting factors of nursing professionalism perceived by nursing students. *Journal of East-West Nursing Research*, 21(1), 10–17. 10.14370/jewnr.2015.21.1.10 (Original work published in Korean)

[bib3] Ayla I. A. Ozyazicioglu N. Atak M., & Surenler S. (2018). Determination of professional values in nursing students. *International Journal of Caring Sciences*, 11(1), 254–261.

[bib4] Bijani M. Tehranineshat B., & Torabizadeh C. (2019). Nurses', nursing students', and nursing instructors' perceptions of professional values: A comparative study. *Nursing Ethics*, 26(3), 870–883. 10.1177/0969733017727153 28905676

[bib5] Briggs C. L. (2011). Engaging students using feature films. *The Journal of Nursing Education*, 50(6), Article 360. 10.3928/01484834-20110519-06 21634327

[bib6] Browne C. Wall P. Batt S., & Bennett R. (2018). Understanding perceptions of nursing professional identity in students entering an Australian undergraduate nursing degree. *Nurse Education in Practice*, 32, 90–96. 10.1016/j.nepr.2018.07.006 30098517

[bib7] Cho J. A., & Kim J. S. (2016). Factors affecting nursing college students' satisfaction with their department. *Journal of the Korea Academia-Industrial Cooperation Society*, 17(4), 587–595. 10.5762/kais.2016.17.4.587 (Original work published in Korean)

[bib8] Cohen J. (1988). *Statistical power analysis for the behavioral sciences* (2nd ed.). Lawrence Erlbaum Associates.

[bib9] Dadich A., & Doloswala N. (2018). What can organisational theory offer knowledge translation in healthcare? A thematic and lexical analysis. *BMC Health Services Research*, 18, Article No. 351. 10.1186/s12913-018-3121-y 29747627 PMC5946475

[bib10] de Braganca A. V., & Nirmala R. (2018). Perceived public image of a nurse and work meaningfulness among nurses. *International Journal of Nursing Education*, 10(3), 1–5. 10.5958/0974-9357.2018.00056.9

[bib11] Edmonds M. L. (2011). Use of film in teaching multiculturalism to future nurse educators. *The Journal of Nursing Education*, 50(9), Article 544. 10.3928/01484834-20110819-02 21866881

[bib12] Emeghebo L. (2012). The image of nursing as perceived by nurses. *Nurse Education Today*, 32(6), e49–e53. 10.1016/j.nedt.2011.10.015 22079480

[bib13] Faul F. Erdfelder E. Lang A. G., & Buchner A. (2007). G*Power 3: A flexible statistical power analysis program for the social, behavioral, and biomedical sciences. *Behavior Research Methods*, 39(2), 175–191. 10.3758/BF03193146 17695343

[bib14] Feng D. Zhao W. Shen S. Chen J., & Li L. (2016). The influence of perceived prejudice on willingness to be a nurse via the mediating effect of satisfaction with major: A cross-sectional study among Chinese male nursing students. *Nurse Education Today*, 42, 69–72. 10.1016/j.nedt.2016.04.012 27237357

[bib15] Ha H. S. (1999). *A study of department satisfaction and school satisfaction of undergraduate students* [Unpublished master's thesis]. Seoul National University, Republic of Korea. (Original work published in Korean)

[bib16] Heldal F. Kongsvik T., & Haland E. (2019). Advancing the status of nursing: Reconstructing professional nursing identity through patient safety work. *BMC Health Services Research*, 19(1), Article No. 418. 10.1186/s12913-019-4222-y 31234881 PMC6591911

[bib17] Kang H. Y. Go M. H. Yang J. J., & Kim S. M. (2003). Nurses' image perceived by academic and vocational high school teachers in Korea. *Journal of Korean Academy of Nursing*, 33(6), 792–801. (Original work published in Korean) 15314397 10.4040/jkan.2003.33.6.792

[bib18] Kantek F. Kaya A., & Gezer N. (2017). The effects of nursing education on professional values: A longitudinal study. *Nurse Education Today*, 58, 43–46. 10.1016/j.nedt.2017.08.004 28866254

[bib19] Kaya H. Işik B. Şenyuva E., & Kaya N. (2017). Personal and professional values held by baccalaureate nursing students. *Nursing Ethics*, 24(6), 716–731. 10.1177/0969733015624488 26822298

[bib20] Kim S. Y. (2014). Effects of biomedical ethics education using movies on biomedical ethics awareness of nursing students. *The Journal of the Korea Contents Association*, 14(7), 281–290. 10.5392/JKCA.2014.14.07.281 (Original work published in Korean)

[bib21] Klemenc-Ketis Z., & Kersnik J. (2011). Using movies to teach professionalism to medical students. *BMC Medical Education*, 11, Article No. 60. 10.1186/1472-6920-11-60 21861900 PMC3180297

[bib22] Kolb D. A. (1984). *Experiential learning: Experience as the source of learning and development*. Prentice-Hall.

[bib23] Korean Nurses Association. (2015). *Nursing association, nursing film festival opening ceremony*. https://www.koreanurse.or.kr/board/board_read.php?board_id=press&member_id=admin&exec=&no=78&category_no=&step=0&tag=&sgroup=76&sfloat=&position=0&mode=&find=stitle&search (Original work published in Korean)

[bib24] Lee D. J. (2004). *The relationships among satisfaction in major, gender identity, and gender stereotypes of male nursing students* [Unpublished master's thesis]. Yonsei University, Seoul, Republic of Korea. (Original work published in Korean)

[bib25] Lim K.-M., & Jo E.-J. (2016). Influence of satisfaction with clinical practice and image of nurses on nursing professionalism of nursing students. *Journal of the Korea Academia-Industrial Cooperation Society*, 17(4), 556–566. 10.5762/KAIS.2016.17.4.556 (Original work published in Korean)

[bib26] Mazhindu D. M. Griffiths L. Pook C. Erskine A. Ellis R., & Smith F. (2016). The nurse match instrument: Exploring professional nursing identity and professional nursing values for future nurse recruitment. *Nursing Education in Practice*, 18, 36–45. 10.1016/j.nepr.2016.03.006 27235564

[bib27] Oh J. Kang J., & de Gagne J. C. (2012). Learning concepts of cinenurducation: An integrative review. *Nurse Education Today*, 32(8), 914–919. 10.1016/j.nedt.2012.03.021 22554554

[bib28] Oh J. Shin H., & de Gagne J. C. D. (2012). QSEN competencies in pre-licensure nursing education and the application to cinenurducation. *Journal of Korean Academic Society of Nursing Education*, 18(3), 474–485. 10.5977/jkasne.2012.18.3.474 (Original work published in Korean)

[bib29] Oh J., & Steefel L. (2016). Nursing students' preferences of strategies surrounding cinenurducation in a first year child growth and development courses: A mixed methods study. *Nurse Education Today*, 36, 342–347. 10.1016/j.nedt.2015.08.019 26343996

[bib30] Oh J.-A. (2010). Review of literature and implication for nursing education: Cinemeducation. *The Journal of Korean Academic Society of Nursing Education*, 16(2), 194–201. (Original work published in Korean)

[bib31] Parandeh A. Khaghanizade M. Mohammadi E., & Mokhtari Nouri J. (2014). Factors influencing development of professional values among nursing students and instructors: A systematic review. *Global Journal of Health Science*, 7(2), 284–293. 10.5539/gjhs.v7n2p284 25716397 PMC4796667

[bib32] Park Y. I. Kim J. A. Ko J.-K. Chung M. S. Bang K.-S. Choe M.-A. Yoo M. S., & Jang H. Y. (2013). An identification study on core nursing competency. *Journal of Korean Academy Society of Nursing Education*, 19(4), 663–674. 10.5977/jkasne.2013.19.4.663 (Original work published in Korean)

[bib33] Pickles D. P. Lacey S., & King L. (2019). Conflict between nursing student's personal beliefs and professional nursing values. *Nursing Ethics*, 26(4), 1087–1100. 10.1177/0969733017738132 29153012

[bib34] Posluszny L., & Hawley D. A. (2017). Comparing professional values of sophomore and senior baccalaureate nursing students. *Journal of Nursing Education*, 56(9), 546–550. 10.3928/01484834-20170817-06 28876441

[bib35] Woo C. H., & Park J. Y. (2017). Specialty satisfaction, positive psychological capital, and nursing professional values in nursing students: A cross-sectional survey. *Nurse Education Today*, 57, 24–28. 10.1016/j.nedt.2017.06.010 28692903

[bib36] Yeun E. J. Kwon Y. M., & Ahn O. H. (2005). Development of a nursing professional values scale. *Journal of Korean Academy Nursing*, 35(6), 1091–1100. (Original work published in Korean) 10.4040/jkan.2005.35.6.1091 16288152

[bib37] Zauderer C. R., & Ganzer C. A. (2011). Cinematic technology: The role of visual learning. *Nurse Educator*, 36(2), 76–79. 10.1097/NNE.0b013e31820b4fbf 21330899

[bib38] Zeppegno P. Gramaglia C. Feggi A. Lombardi A., & Torre E. (2015). The effectiveness of a new approach using movies in the training of medical students. *Perspectives on Medical Education*, 4(5), 261–263. 10.1007/s40037-015-0208-6 26346496 PMC4602017

